# Relative Suffix Trees

**DOI:** 10.1093/comjnl/bxx108

**Published:** 2017-11-21

**Authors:** Andrea Farruggia, Travis Gagie, Gonzalo Navarro, Simon J Puglisi, Jouni Sirén

**Affiliations:** 1 Department of Computer Science, University of Pisa, Largo Bruno Pontecorvo 3, 56127 Pisa PI, Italy; 2 CeBiB—Center for Biotechnology and Bioengineering, Santiago, Chile; 3 Escuela de Informática y Telecomunicaciones, Diego Portales University, Ejército 441, Santiago, Chile; 4 Department of Computer Science, University of Chile, Beauchef 851, Santiago, Chile; 5 Department of Computer Science, University of Helsinki, Helsinki, Finland; 6 Wellcome Trust Sanger Institute, Hinxton CB10 1SA, UK

**Keywords:** suffix trees, compressed text indexing, repetitive collections

## Abstract

Suffix trees are one of the most versatile data structures in stringology, with many applications in bioinformatics. Their main drawback is their size, which can be tens of times larger than the input sequence. Much effort has been put into reducing the space usage, leading ultimately to compressed suffix trees. These compressed data structures can efficiently simulate the suffix tree, while using space proportional to a compressed representation of the sequence. In this work, we take a new approach to compressed suffix trees for repetitive sequence collections, such as collections of individual genomes. We compress the suffix trees of individual sequences relative to the suffix tree of a reference sequence. These relative data structures provide competitive time/space trade-offs, being almost as small as the smallest compressed suffix trees for repetitive collections, and competitive in time with the largest and fastest compressed suffix trees.

## 1. INTRODUCTION

The *suffix tree* [1] is one of the most powerful bioinformatic tools to answer complex queries on DNA and protein sequences [2–4]. A serious problem that hampers its wider use on large genome sequences is its size, which may be 10–20 bytes per character. In addition, the non-local access patterns required by most interesting problems solved with suffix trees complicate secondary-memory deployments. This problem has led to numerous efforts to reduce the size of suffix trees by representing them using *compressed data structures* [5–17], leading to *compressed suffix trees* (CST). Currently, the smallest CST is the so-called *fully compressed suffix tree* (FCST) [10, 14], which uses 5 bits per character (bpc) for DNA sequences, but takes milliseconds to simulate suffix tree navigation operations. In the other extreme, Sadakane’s CST [5, 11] uses about 12 bpc and operates in microseconds, and even nanoseconds for the simplest operations.

A space usage of 12 bpc may seem reasonable to handle, for example, one human genome, which has about 3.1 billion bases: it can be operated within a RAM of 4.5 GB (the representation contains the sequence as well). However, as the price of sequencing has fallen, sequencing the genomes of a large number of individuals has become a routine activity. The 1000 *Genomes Project* [18] sequenced the genomes of several thousand humans, while newer projects can be orders of magnitude larger. This has made the development of techniques for storing and analyzing huge amounts of sequence data flourish.

Just storing 1000 human genomes using a 12 bpc CST requires almost 4.5 TB, which is much more than the amount of memory available in a commodity server. Assuming that a single server has 256 GB of memory, we would need a cluster of 18 servers to handle such a collection of CSTs (compared with over 100 with classical suffix tree implementations!). With the smaller (and much slower) FCST, this would drop to 7–8 servers. It is clear that further space reductions in the representation of CST would lead to reductions in hardware, communication and energy costs when implementing complex searches over large genomic databases.

An important characteristic of those large genome databases is that they usually consist of the genomes of individuals of the same or closely related species. This implies that the collections are highly *repetitive*, that is, each genome can be obtained by concatenating a relatively small number of substrings of other genomes and adding a few new characters. When repetitiveness is considered, much higher compression rates can be obtained in CST. For example, it is possible to reduce the space to 1–2 bpc (albeit with operation times in the milliseconds) [13], or to 2–3 bpc with operation times in the microseconds [15]. Using 2 bpc, our 1000 genomes could be handled with just three servers with 256 GB of memory.

Compression algorithms best capture repetitiveness by using *grammar-based* compression or *Lempel–Ziv* compression.^1^ In the first case [19, 20], one finds a context-free grammar that generates (only) the text collection. Rather than compressing the text directly, the current CSTs for repetitive collections [13, 15] apply grammar-based compression on the data structures that simulate the suffix tree. Grammar-based compression yields relatively easy direct access to the compressed sequence [21], which makes it attractive compared to Lempel–Ziv compression [22], despite the latter generally using less space.

Lempel–Ziv compression cuts the collection into *phrases*, each of which has already appeared earlier in the collection. To extract the content of a phrase, one may have to recursively extract the content at that earlier position, following a possibly long chain of indirections. So far, the indexes built on Lempel–Ziv compression [23] or on combinations of Lempel–Ziv and grammar-based compression [24–26] support only pattern matching, which is just one of the wide range of functionalities offered by suffix trees. The high cost to access the data at random positions lies at the heart of the research on indexes built on Lempel–Ziv compression.

A simple way out of this limitation is the so-called *relative Lempel–Ziv* (RLZ) compression [27], where one of the sequences is represented in plain form and the others can only take phrases from that *reference sequence*. This enables immediate access for the symbols inside any copied phrase (as no transitive referencing exists) and, at least, if a good reference sequence has been found, offers compression competitive with the classical Lempel–Ziv. In our case, taking any random genome per species as the reference is good enough; more sophisticated techniques have been studied [28–30]. Structures for direct access [31, 32] and even for pattern matching [33] have been developed on top of RLZ.

Another approach to compressing a repetitive collection while supporting interesting queries is to build an automaton that accepts the sequences in the collection, and then index the state diagram as an directed acyclic graph (DAG); see, for example, [34–36] for recent discussions. The first data structure to take this approach was the generalized compressed suffix array (GCSA) [37, 36], which was designed for pangenomics so queries can return information about sequences not in the collection but that can be obtained from those in the collection by recombination.

The FM-index of an alignment (FMA) [38, 39] is similar to the GCSA but indexes only the sequences in the collection: whereas the GCSA conceptually embeds the automaton in a de Bruijn graph, the FMA embeds it in a colored de Bruijn graph [40], preserving its specificity. Both the GCSA and the FMA are practical but neither support the full functionality of a suffix tree. The precursor to the FMA, the suffix tree of an alignment (STA) [41, 42], allows certain disjunctions in the suffix tree’s edge labels in order to reduce the size of the tree while maintaining its functionality. Unlike the FMA, however, the STA has not been implemented. Both the STA and the FMA divide the sequences in the collection into regions of variation and conserved regions, and depend on the conserved regions being long enough that they can be distinguished from each other and the variations. This dependency makes these structures vulnerable to even a small change in even one sequence to an otherwise-conserved region, which could hamper their scalability.

### 1.1. One general 𝖢𝖲𝖳 or many individual 𝖢𝖲𝖳 s

It is important to note that the existing techniques to reduce the space of a collection of suffix trees on similar texts build a structure that indexes the collection *as a whole*, which is similar to concatenating all the texts of the collection and building a single suffix tree on the concatenation. As such, these structures do not provide the same functionality of having an individual CST of each sequence.

Exploiting the repetitiveness of a collection while retaining separate index structures for each text has only been achieved for a simpler pattern-matching index, the *suffix array* (SA) [43], by means of the so-called relative FM-indexes (FMIs) [44]. The SA is a component of the suffix tree.

Depending on the application, we may actually need a single 𝖢𝖲𝖳 for the whole collection, or one for each sequence. In bioinformatics, a single 𝖢𝖲𝖳 is more appropriate for search and discovery of motifs across a whole population, for example, by looking for approximate occurrences of a certain sequence in the genomes of the population or by discovering significant sequences that appear in many individuals. Other bioinformatic problems, for example related to the study of diseases, inheritance patterns or forensics, boil down to searching or discovering patterns in the genomes of individuals, by finding common approximate subsequences between two genomes, or looking for specific motifs or discovering certain patterns in a single genome.

An example of recent research making use of the relative storage of individual genomic datasets is how Muggli et al. [45] (see also [46, 47]) adapted relative FMIs to an FMI variant that Bowe et al. [48] had described for de Bruijn graphs, thus obtaining a space-efficient implementation of Iqbal *et al*.’s [49] colored de Bruijn graphs. These overlay de Bruijn graphs for many individuals to represent genetic variation in a population.

### 1.2. Our contribution

In this paper, we develop a CST for repetitive collections by augmenting the relative FMI with structures based on RLZ. This turns out to be the first CST representation that takes advantage of the repetitiveness of the texts in a collection while at the same time offering an individual CST for each such text. Besides retaining the original functionality, such an approach greatly simplifies inserting and deleting texts in the collection and implementing the index in distributed form.

Our compressed suffix tree, called relative suffix tree (RST), follows a trend of CSTs [6–9, 11, 13] that use only a SA and an array with the length of the longest common prefix (LCP) between each suffix and the previous one in lexicographic order (called LCP). We use the relative FMI as our SA, and compress LCP using RLZ. On top of the RLZ phrases we build a tree of range minima that enables fast range minimum queries, as well as next- and previous-smaller-value queries, on LCP [13]. All the CST functionality is built on those queries [6]. Our main algorithmic contribution is this RLZ-based representation of the LCP array with the required extra functionality.

On a collection of human genomes, our RST achieves less than 3 bpc and operates within microseconds. This performance is comparable to that of a previous CST [15] (as explained, however, the RST provides a different functionality because it retains the individual CSTs).

## 2. BACKGROUND

A *string*$S[1,n]=s_{1},\ldots ,s_{n}$ is a sequence of *characters* over an *alphabet*Σ={1,…,σ}. For indexing purposes, we often consider *text* strings T[1,n] that are terminated by an *endmarker*T[n]=$=0 not occurring elsewhere in the text. *Binary* sequences are sequences over the alphabet {0,1}. If B[1,n] is a binary sequence, its *complement* is binary sequence $\overset{¯}{B}[1,n]$, with $\overset{¯}{B}\left[i\right]=1-B\left[i\right]$.

For any binary sequence B[1,n], we define the *subsequence*S[B] of string S[1,n] as the concatenation of the characters $s_{i}$ with B[i]=1. The complement $\overset{¯}{S}\left[B\right]$ of subsequence S[B] is the subsequence $S\left[\overset{¯}{B}\right]$. Contiguous subsequences S[i,j] are called *substrings*. Substrings of the form S[1,j] and S[i,n], i,j∈[1,n], are called *prefixes* and *suffixes*, respectively. We define the *lexicographic order* among strings in the usual way.

### 2.1. Full-text indexes

The *suffix tree* (ST) [1] of text T is a tree containing the suffixes of T, with unary paths compacted into single edges. Because the degree of every internal node is at least two, there can be at most 2n−1 nodes, and the suffix tree can be stored in 𝖮(nlogn) bits. In practice, this is at least 10n bytes for small texts [50], and more for large texts as the pointers grow larger. If v is a node of a suffix tree, we write π(v) to denote the concatenation of the labels of the path from the root to v.

SAs [43] were introduced as a space-efficient alternative to suffix trees. The SA ${\mathrm{𝖲𝖠}}_{T}[1,n]$ of text T is an array of pointers to the suffixes of the text in lexicographic order.^2^ In its basic form, the SA requires nlogn bits in addition to the text, but its functionality is more limited than that of the suffix tree. In addition to the SA, many algorithms also use the *inverse SA*𝖨𝖲𝖠[1,n], with 𝖲𝖠[𝖨𝖲𝖠[i]]=i for all i.

Let $\mathrm{𝗅𝖼𝗉}(S_{1},S_{2})$ be the length of the (LCP) of strings $S_{1}$ and $S_{2}$. The LCP*array* [43] 𝖫𝖢𝖯[1,n] of text T stores the LCP lengths for lexicographically adjacent suffixes of T as 𝖫𝖢𝖯[i]=𝗅𝖼𝗉(T[𝖲𝖠[i−1],n],T[𝖲𝖠[i],n]) (with 𝖫𝖢𝖯[1]=0). Let v be an internal node of the suffix tree, ℓ=∣π(v)∣ the *string depth* of node v, and 𝖲𝖠[sp,ep] the corresponding SA interval. The following properties hold for the *lcp-interval*𝖫𝖢𝖯[sp,ep]: (i) 𝖫𝖢𝖯[sp]<ℓ; (ii) 𝖫𝖢𝖯[i]≥ℓ for all sp<i≤ep; (iii) 𝖫𝖢𝖯[i]=ℓ for at least one sp<i≤ep; and (iv) 𝖫𝖢𝖯[ep+1]<ℓ [51].

Abouelhoda, Kurtz and Ohlebusch [51] showed how traversals on the suffix tree could be simulated using the SA, the LCP array, and a representation of the suffix tree topology based on lcp-intervals, paving the way for more space-efficient suffix tree representations.

### 2.2. Compressed text indexes

Data structures supporting rank and select queries over sequences are the main building blocks of compressed text indexes. If S is a sequence, we define ${\mathrm{𝗋𝖺𝗇𝗄}}_{c}(S,i)$ as the number of occurrences of character c in the prefix S[1,i], while ${\mathrm{𝗌𝖾𝗅𝖾𝖼𝗍}}_{c}(S,j)$ is the position of the occurrence of rank j in sequence S. A *bitvector* is a representation of a binary sequence supporting fast rank and select queries. *Wavelet trees* (WT) [52] use bitvectors to support rank and select on general sequences.

The *Burrows–Wheeler transform* (BWT) [53] is a reversible permutation 𝖡𝖶𝖳[1,n] of text T. It is defined as 𝖡𝖶𝖳[i]=T[𝖲𝖠[i]−1] (with 𝖡𝖶𝖳[i]=T[n] if 𝖲𝖠[i]=1). Originally intended for data compression, the BWT has been widely used in space-efficient text indexes, because it shares the combinatorial structure of the suffix tree and the SA.

Let LF be a function such that 𝖲𝖠[𝖫𝖥(i)]=𝖲𝖠[i]−1 (with 𝖲𝖠[𝖫𝖥(i)]=n if 𝖲𝖠[i]=1). We can compute it as $\mathrm{𝖫𝖥}\left(i\right)=𝖢[\mathrm{𝖡𝖶𝖳}\left[i\right]]+{\mathrm{𝗋𝖺𝗇𝗄}}_{\mathrm{𝖡𝖶𝖳}\left[i\right]}(\mathrm{𝖡𝖶𝖳},i)$, where 𝖢[c] is the number of occurrences of characters with lexicographical values smaller than c in BWT. The inverse function of LF is Ψ, with $\Psi \left(i\right)={\mathrm{𝗌𝖾𝗅𝖾𝖼𝗍}}_{c}(\mathrm{𝖡𝖶𝖳},i-𝖢\left[c\right])$, where c is the largest character value with 𝖢[c]<i. With functions Ψ and LF, we can move forward and backward in the text, while maintaining the lexicographic rank of the current suffix. If the sequence S is not evident from the context, we write ${\mathrm{𝖫𝖥}}_{S}$ and $\Psi_{S}$.

*Compressed SAs* (CSA) [54–56] are text indexes supporting a functionality similar to the SA. This includes the following queries: (i) 𝖿𝗂𝗇𝖽(P)=[sp,ep] determines the lexicographic range of suffixes starting with *pattern*P[1,ℓ]; (ii) 𝗅𝗈𝖼𝖺𝗍𝖾(sp,ep)=𝖲𝖠[sp,ep] returns the starting positions of these suffixes; and (iii) 𝖾𝗑𝗍𝗋𝖺𝖼𝗍(i,j)=T[i,j] extracts substrings of the text. In practice, the find performance of CSAs can be competitive with SAs, while locate queries are orders of magnitude slower [57]. Typical index sizes are less than the size of the uncompressed text.

The FMI [55] is a common type of CSA. A typical implementation [58] stores the BWT in a wavelet tree [52]. The index implements find queries via *backward searching*. Let [sp,ep] be the lexicographic range of the suffixes of the text starting with suffix P[i+1,ℓ] of the pattern. We can find the range matching suffix P[i,ℓ] with a generalization of function LF as

$$\begin{array}{ccc} \mathrm{𝖫𝖥}([\mathrm{sp},\mathrm{ep}],P\left[i\right]) & = & [𝖢[P\left[i\right]]+{\mathrm{𝗋𝖺𝗇𝗄}}_{P\left[i\right]}(\mathrm{𝖡𝖶𝖳},\mathrm{sp}-1)+1,\\ & & 𝖢[P\left[i\right]]+{\mathrm{𝗋𝖺𝗇𝗄}}_{P\left[i\right]}(\mathrm{𝖡𝖶𝖳},\mathrm{ep})]. \end{array}$$

We support locate queries by *sampling* some SA pointers. If we want to determine a value 𝖲𝖠[i] that has not been sampled, we can compute it as 𝖲𝖠[i]=𝖲𝖠[j]+k, where 𝖲𝖠[j] is a sampled pointer found by iterating LFk times, starting from position i. Given *sample interval*d, the samples can be chosen in *suffix order*, sampling 𝖲𝖠[i] at positions divisible by d, or in *text order*, sampling T[i] at positions divisible by d and marking the sampled SA positions in a bitvector. Suffix-order sampling requires less space, often resulting in better time/space trade-offs in practice, while text-order sampling guarantees better worst-case performance. We also sample the ISA pointers for extract queries. To extract T[i,j], we find the nearest sampled pointer after T[j], and traverse backwards to T[i] with function LF.

CST [5] are compressed text indexes supporting the full functionality of a suffix tree (see Table 1). They combine a CSA, a compressed representation of the LCP array, and a compressed representation of suffix tree topology. For the LCP array, there are several common representations:

- LCP-byte [51] stores the LCP array as a byte array. If 𝖫𝖢𝖯[i]<255, the LCP value is stored in the byte array. Larger values are marked with a 255 in the byte array and stored separately. As many texts produce small LCP values, LCP-byte usually requires n to 1.5n bytes of space.
- We can store the LCP array by using variable-length codes. LCP-dac uses *directly addressable codes* [59] for the purpose, resulting in a structure that is typically somewhat smaller and somewhat slower than LCP-byte.
- The *permuted*LCP (PLCP) *array* [5] 𝖯𝖫𝖢𝖯[1,n] is the LCP array stored in text order and used as 𝖫𝖢𝖯[i]=𝖯𝖫𝖢𝖯[𝖲𝖠[i]]. Because 𝖯𝖫𝖢𝖯[i+1]≥𝖯𝖫𝖢𝖯[i]−1, the array can be stored as a bitvector of length 2n in 2n+𝗈(n) bits. If the text is repetitive, run-length encoding can be used to compress the bitvector to take even less space [6]. Because accessing PLCP uses locate, it is much slower than the above two encodings.

**Table 1.** Typical compressed suffix tree operations.

Operation	Description
𝖱𝗈𝗈𝗍()	The root of the tree
𝖫𝖾𝖺𝖿(v)	Is node v a leaf?
𝖠𝗇𝖼𝖾𝗌𝗍𝗈𝗋(v,w)	Is node v an ancestor of node w?
𝖢𝗈𝗎𝗇𝗍(v)	Number of leaves in the subtree with v as the root
𝖫𝗈𝖼𝖺𝗍𝖾(v)	Pointer to the suffix corresponding to leaf v
𝖯𝖺𝗋𝖾𝗇𝗍(v)	The parent of node v
𝖥𝖢𝗁𝗂𝗅𝖽(v)	The first child of node v in alphabetic order
𝖭𝖲𝗂𝖻𝗅𝗂𝗇𝗀(v)	The next sibling of node v in alphabetic order
𝖫𝖢𝖠(v,w)	The lowest common ancestor of nodes v and w
𝖲𝖣𝖾𝗉𝗍𝗁(v)	*String depth*: length ℓ=∣π(v)∣ of the label from the root to node v
𝖳𝖣𝖾𝗉𝗍𝗁(v)	*Tree depth*: the depth of node v in the suffix tree
${\mathrm{𝖫𝖠𝖰}}_{S}(v,d)$	The highest ancestor of node v with string depth at least d
${\mathrm{𝖫𝖠𝖰}}_{T}(v,d)$	The ancestor of node v with tree depth d
𝖲𝖫𝗂𝗇𝗄(v)	*Suffix link*: Node w such that π(v)=cπ(w) for a character c∈Σ
${\mathrm{𝖲𝖫𝗂𝗇𝗄}}^{k}\left(v\right)$	Suffix link iterated k times
𝖢𝗁𝗂𝗅𝖽(v,c)	The child of node v with edge label starting with character c
𝖫𝖾𝗍𝗍𝖾𝗋(v,i)	The character π(v)[i]

Suffix tree topology representations are the main difference between the various CST proposals. While the CSAs and the LCP arrays are interchangeable, the tree representation determines how various suffix tree operations are implemented. There are three main families of CST:

- *Sadakane*’*s compressed suffix tree* (CST-Sada) [5] uses a *balanced parentheses* representation for the tree. Each node is encoded as an opening parenthesis, followed by the encodings of its children and a closing parenthesis. This can be encoded as a bitvector of length $2n^{\prime }$, where $n^{\prime }$ is the number of nodes, requiring up to 4n+𝗈(n) bits. CST-Sada tends to be larger and faster than the other compressed suffix trees [11, 13].
- The *fully compressed suffix tree* (FCST) of Russo et al. [10, 14] aims to use as little space as possible. It does not require an LCP array at all, and stores a balanced parentheses representation for a sampled subset of suffix tree nodes in 𝗈(n) bits. Unsampled nodes are retrieved by following suffix links. FCST is smaller and much slower than the other CST [10, 13].
- Fischer, Mäkinen and Navarro [6] proposed an intermediate representation, CST-NPR, based on lcp-intervals. Tree navigation is handled by searching for the values defining the lcp-intervals. *Range minimum queries*𝗋𝗆𝗊(sp,ep) find the leftmost minimal value in 𝖫𝖢𝖯[sp,ep], while *next/previous smaller value* queries 𝗇𝗌𝗏(i)/𝗉𝗌𝗏(i) find the next/previous LCP value smaller than 𝖫𝖢𝖯[i]. After the improvements by various authors [7–9, 11, 13], the CST-NPR is perhaps the most practical compressed suffix tree.

For typical texts and component choices, the size of CST ranges from the 1.5n to 3n bytes of CST-Sada to the 0.5n to n bytes of FCST [11, 13]. There are also some CST variants for repetitive texts, such as versioned document collections and collections of individual genomes. Abeliuk et al. [13] developed a variant of CST-NPR that can sometimes be smaller than n bits, while achieving performance similar to the FCST. Navarro and Ordez [15] used grammar-based compression for the tree representation of CST-Sada. The resulting compressed suffix tree (GCT) requires slightly more space than the CST-NPR of Abeliuk et al., while being closer to the non-repetitive CST-Sada and CST-NPR in performance.

### 2.3. Relative Lempel–Ziv

RLZ parsing [27] compresses *target* sequence S relative to *reference* sequence R. The target sequence is represented as a concatenation of z*phrases*$w_{i}=(p_{i},\ell_{i},c_{i})$, where $p_{i}$ is the starting position of the phrase in the reference, $\ell_{i}$ is the length of the copied substring and $c_{i}$ is the *mismatch* character. If phrase $w_{i}$ starts from position $p^{\prime }$ in the target, then $S[p^{\prime },p^{\prime }+\ell_{i}-1]=R[p_{i},p_{i}+\ell_{i}-1]$ and $S[p^{\prime }+\ell_{i}]=c_{i}$.

The shortest RLZ parsing of the target sequence can be found in (essentially) linear time. The algorithm builds a CSA for the reverse of the reference sequence, and then parses the target sequence greedily by using backward searching. If the edit distance between the reference and the target is s, we need at most s phrases to represent the target sequence. On the other hand, because the relative order of the phrases can be different in sequences R and S, the edit distance can be much larger than the number of phrases in the shortest RLZ parsing.

In a straightforward implementation, the *phrase pointers*$p_{i}$ and the mismatch characters $c_{i}$ can be stored in arrays $W_{p}$ and $W_{c}$. These arrays take zlog∣R∣ and zlogσ bits. To support random access to the target sequence, we can encode phrase lengths as a bitvector $W_{\ell }$ of length ∣S∣ [27]: we set $W_{\ell }\left[j\right]=1$ if S[j] is the first character of a phrase. The bitvector requires $z\log\frac{n}{z}+𝖮\left(z\right)$ bits if we use the sdarray representation [60]. To extract S[j], we first determine the phrase $w_{i}$, with $i={\mathrm{𝗋𝖺𝗇𝗄}}_{1}(W_{\ell },j)$. If $W_{\ell }[j+1]=1$, we return the mismatch character $W_{c}\left[i\right]$. Otherwise we determine the phrase offset with a select query, and return the character $R[W_{p}\left[i\right]+j\;-{\mathrm{𝗌𝖾𝗅𝖾𝖼𝗍}}_{1}(W_{\ell },i)]$.

Ferrada et al. [32] showed how, by using *relative pointers* instead of absolute pointers, we can avoid the use of select queries. They also achieved better compression of DNA collections, in which most of the differences between the target sequences and the reference sequence are single-character *substitutions*. By setting $W_{r}\left[i\right]=p_{i}-{\mathrm{𝗌𝖾𝗅𝖾𝖼𝗍}}_{1}(W_{\ell },i)$, the general case simplifies to $S\left[j\right]=R[W_{r}\left[i\right]+j]$. If most of the differences are single-character substitutions, $p_{i+1}$ will often be $p_{i}+\ell_{i}+1$. This corresponds to $W_{r}[i+1]=W_{r}\left[i\right]$ with relative pointers, making *run-length encoding* of the pointer array worthwhile.

When we sort the suffixes in lexicographic order, substitutions in the text move suffixes around, creating *insertions* and *deletions* in the SA and related structures. In the LCP array, an insertion or deletion affecting 𝖫𝖢𝖯[i] can also change the value of 𝖫𝖢𝖯[i+1]. Hence, RLZ with relative pointers is not enough to compress the LCP array.

Cox et al. [61] modified Ferrada et al.’s version of RLZ to handle other small variations in addition to single-character substitutions. After adding a phrase to the parse, we look ahead a bounded number of positions to find potential phrases with a relative pointer $W_{r}\left[i\right]$ close to the previous *explicit* relative pointer $W_{r}\left[j\right]$. If we can find a sufficiently long phrase this way, we encode the pointer *differentially* as $W_{r}\left[i\right]-W_{r}\left[j\right]$. Otherwise we store $W_{r}\left[i\right]$ explicitly. We can then save space by storing the differential pointers separately using less bits per pointer. Because there can be multiple mismatch characters between phrases i and i+1, we also need a prefix-sum data structure L for finding the range $W_{c}[a,b]$ containing the mismatches. Cox et al. showed that their approach compresses both DNA sequences and LCP arrays better than Ferrada et al.’s version, albeit with slightly slower random access. We refer the reader to their paper for more details of their implementation.

## 3. RELATIVE FMI

The *relative FMI* (RFM) [44] is a compressed SA of a sequence relative to the CSA of another sequence. The index is based on approximating the *longest common subsequence* (LCS) of ${\mathrm{𝖡𝖶𝖳}}_{R}$ and ${\mathrm{𝖡𝖶𝖳}}_{S}$, where R is the reference sequence and S is the target sequence, and storing several structures based on the common subsequence. Given a representation of ${\mathrm{𝖡𝖶𝖳}}_{R}$ supporting rank and select, we can use the relative index ${\mathrm{𝖱𝖥𝖬}}_{S|R}$ to simulate rank and select on ${\mathrm{𝖡𝖶𝖳}}_{S}$.

In this section, we describe the relative FMI using the notation and the terminology of this paper. We also give an explicit description of the locate and extract functionality, which was not included in the original paper. Finally, we describe a more space-efficient variant of the algorithm for building a relative FMI with full functionality.

### 3.1. Basic index

Assume that we have found a long common subsequence of sequences X and Y. We call positions X[i] and Y[j]*lcs-positions*, if they are in the common subsequence. If $B_{X}$ and $B_{Y}$ are the binary sequences marking the common subsequence ($X[{\mathrm{𝗌𝖾𝗅𝖾𝖼𝗍}}_{1}(B_{X},i)]=Y[{\mathrm{𝗌𝖾𝗅𝖾𝖼𝗍}}_{1}(B_{Y},i)]$), we can move between lcs-positions in the two sequences with rank and select operations. If X[i] is an lcs-position, the corresponding position in sequence Y is $Y[{\mathrm{𝗌𝖾𝗅𝖾𝖼𝗍}}_{1}(B_{Y},{\mathrm{𝗋𝖺𝗇𝗄}}_{1}(B_{X},i))]$. We denote this pair of *lcs-bitvectors*$\mathrm{𝖠𝗅𝗂𝗀𝗇}(X,Y)=\langle B_{X},B_{Y}\rangle$.

In its most basic form, the relative FMI ${\mathrm{𝖱𝖥𝖬}}_{S|R}$ only supports find queries by simulating rank queries on ${\mathrm{𝖡𝖶𝖳}}_{S}$. It does this by storing $\mathrm{𝖠𝗅𝗂𝗀𝗇}({\mathrm{𝖡𝖶𝖳}}_{R},{\mathrm{𝖡𝖶𝖳}}_{S})$ and the complements (subsequences of non-aligned characters) $\overset{¯}{\mathrm{𝖠𝗅𝗂𝗀𝗇}}\left({\mathrm{𝖡𝖶𝖳}}_{R}\right)$ and $\overset{¯}{\mathrm{𝖠𝗅𝗂𝗀𝗇}}\left({\mathrm{𝖡𝖶𝖳}}_{S}\right)$. The lcs-bitvectors are compressed using *entropy-based compression* [62], while the complements are stored in structures similar to the reference ${\mathrm{𝖡𝖶𝖳}}_{R}$.

To compute ${\mathrm{𝗋𝖺𝗇𝗄}}_{c}({\mathrm{𝖡𝖶𝖳}}_{S},i)$, we first determine the number of lcs-positions in ${\mathrm{𝖡𝖶𝖳}}_{S}$ up to position S[i] with $k\;={\mathrm{𝗋𝖺𝗇𝗄}}_{1}(B_{{\mathrm{𝖡𝖶𝖳}}_{S}},i)$. Then we find the lcs-position k in ${\mathrm{𝖡𝖶𝖳}}_{R}$ with $j={\mathrm{𝗌𝖾𝗅𝖾𝖼𝗍}}_{1}(B_{{\mathrm{𝖡𝖶𝖳}}_{R}},k)$. With these positions, we can compute

$$\begin{array}{ccc} {\mathrm{𝗋𝖺𝗇𝗄}}_{c}({\mathrm{𝖡𝖶𝖳}}_{S},i) & = & {\mathrm{𝗋𝖺𝗇𝗄}}_{c}({\mathrm{𝖡𝖶𝖳}}_{R},j)\\ & & -{\mathrm{𝗋𝖺𝗇𝗄}}_{c}(\overset{¯}{\mathrm{𝖠𝗅𝗂𝗀𝗇}}\left({\mathrm{𝖡𝖶𝖳}}_{R}\right),j-k)\\ & & +{\mathrm{𝗋𝖺𝗇𝗄}}_{c}(\overset{¯}{\mathrm{𝖠𝗅𝗂𝗀𝗇}}\left({\mathrm{𝖡𝖶𝖳}}_{S}\right),i-k). \end{array}$$

### 3.2. Relative select

We can implement the entire functionality of a CSA with rank queries on the BWT. However, if we use the CSA in a compressed suffix tree, we also need select queries to support *forward searching* with Ψ and 𝖢𝗁𝗂𝗅𝖽 queries. We can always implement select queries by binary searching with rank queries, but the result will be much slower than the rank queries.

A faster alternative to support select queries in the relative FMI is to build a *relative select* structure rselect [63]. Let ${𝖥}_{X}$ be a sequence consisting of the characters of sequence X in sorted order. Alternatively, ${𝖥}_{X}$ is a sequence such that ${𝖥}_{X}\left[i\right]\;={\mathrm{𝖡𝖶𝖳}}_{X}[\Psi_{X}\left(i\right)]$. The relative select structure consists of bitvectors $\mathrm{𝖠𝗅𝗂𝗀𝗇}({𝖥}_{R},{𝖥}_{S})$, where $B_{{𝖥}_{R}}\left[i\right]=B_{{\mathrm{𝖡𝖶𝖳}}_{R}}[\Psi_{R}\left(i\right)]$ and $B_{{𝖥}_{S}}\left[i\right]\;=B_{{\mathrm{𝖡𝖶𝖳}}_{S}}[\Psi_{S}\left(i\right)]$, as well as the C array ${𝖢}_{\mathrm{𝖫𝖢𝖲}}$ for the common subsequence.

To compute ${\mathrm{𝗌𝖾𝗅𝖾𝖼𝗍}}_{c}({\mathrm{𝖡𝖶𝖳}}_{S},i)$, we first determine how many of the first i occurrences of character c are lcs-positions with $k={\mathrm{𝗋𝖺𝗇𝗄}}_{1}(B_{{𝖥}_{S}},{𝖢}_{{\mathrm{𝖡𝖶𝖳}}_{S}}\left[c\right]+i)-{𝖢}_{\mathrm{𝖫𝖢𝖲}}\left[c\right]$. Then we check from bit $B_{{𝖥}_{S}}[{𝖢}_{{\mathrm{𝖡𝖶𝖳}}_{S}}\left[c\right]+i]$ whether the occurrence we are looking for is an lcs-position or not. If it is, we find the position in ${\mathrm{𝖡𝖶𝖳}}_{R}$ as $j={\mathrm{𝗌𝖾𝗅𝖾𝖼𝗍}}_{c}({\mathrm{𝖡𝖶𝖳}}_{R},{\mathrm{𝗌𝖾𝗅𝖾𝖼𝗍}}_{1}(B_{{𝖥}_{R}},{𝖢}_{\mathrm{𝖫𝖢𝖲}}\left[c\right]+k)-{𝖢}_{R}\left[c\right])$, and then map j to ${\mathrm{𝗌𝖾𝗅𝖾𝖼𝗍}}_{c}({\mathrm{𝖡𝖶𝖳}}_{S},i)$ by using $\mathrm{𝖠𝗅𝗂𝗀𝗇}({\mathrm{𝖡𝖶𝖳}}_{R},{\mathrm{𝖡𝖶𝖳}}_{S})$. Otherwise we find the occurrence in $\overset{¯}{\mathrm{𝖠𝗅𝗂𝗀𝗇}}\left({\mathrm{𝖡𝖶𝖳}}_{S}\right)$ with $j={\mathrm{𝗌𝖾𝗅𝖾𝖼𝗍}}_{c}(\overset{¯}{\mathrm{𝖠𝗅𝗂𝗀𝗇}}\left({\mathrm{𝖡𝖶𝖳}}_{S}\right),i-k)$, and return ${\mathrm{𝗌𝖾𝗅𝖾𝖼𝗍}}_{c}({\mathrm{𝖡𝖶𝖳}}_{S},i)\;={\mathrm{𝗌𝖾𝗅𝖾𝖼𝗍}}_{0}(B_{{\mathrm{𝖡𝖶𝖳}}_{S}},j)$.

### 3.3. Full functionality

If we want the relative FMI to support locate and extract queries, we cannot build it from any common subsequence of ${\mathrm{𝖡𝖶𝖳}}_{R}$ and ${\mathrm{𝖡𝖶𝖳}}_{S}$. We need a *bwt-invariant subsequence* [44], where the alignment of the BWTs is also an alignment of the original sequences.

**Definition 1**

*Let*X*be a common subsequence of*${\mathrm{𝖡𝖶𝖳}}_{R}$*and*${\mathrm{𝖡𝖶𝖳}}_{S}$, *and let*${\mathrm{𝖡𝖶𝖳}}_{R}\left[i_{R}\right]$*and*${\mathrm{𝖡𝖶𝖳}}_{S}\left[i_{S}\right]$*be the lcs-positions corresponding to*X[i]. *Subsequence X is bwt-invariant if*

$${\mathrm{𝖲𝖠}}_{R}\left[i_{R}\right]<{\mathrm{𝖲𝖠}}_{R}\left[j_{R}\right]\;⟺\;{\mathrm{𝖲𝖠}}_{S}\left[i_{S}\right]<{\mathrm{𝖲𝖠}}_{S}\left[j_{S}\right]$$

*for all positions*i,j∈{1,…,∣X∣}.

In addition to the structures already mentioned, the full relative FMI has another pair of lcs-bitvectors, 𝖠𝗅𝗂𝗀𝗇(R,S), which marks the bwt-invariant subsequence in the original sequences. If ${\mathrm{𝖡𝖶𝖳}}_{R}\left[i_{R}\right]$ and ${\mathrm{𝖡𝖶𝖳}}_{S}\left[i_{S}\right]$ are lcs-positions, we set $B_{R}[{\mathrm{𝖲𝖠}}_{R}\left[i_{R}\right]-1]=1$ and $B_{S}[{\mathrm{𝖲𝖠}}_{S}\left[i_{S}\right]-1]=1$.^3^

To compute the answer to a 𝗅𝗈𝖼𝖺𝗍𝖾(i) query, we start by iterating ${\mathrm{𝖡𝖶𝖳}}_{S}$ backwards with LF queries, until we find an lcs-position ${\mathrm{𝖡𝖶𝖳}}_{S}\left[i^{\prime }\right]$ after k steps. Then we map position $i^{\prime }$ to the corresponding position $j^{\prime }$ in ${\mathrm{𝖡𝖶𝖳}}_{R}$ by using $\mathrm{𝖠𝗅𝗂𝗀𝗇}({\mathrm{𝖡𝖶𝖳}}_{R},{\mathrm{𝖡𝖶𝖳}}_{S})$. Finally, we determine ${\mathrm{𝖲𝖠}}_{R}\left[j^{\prime }\right]$ with a locate query in the reference index, and map the result to ${\mathrm{𝖲𝖠}}_{S}\left[i^{\prime }\right]$ by using 𝖠𝗅𝗂𝗀𝗇(R,S).^4^ The result of the 𝗅𝗈𝖼𝖺𝗍𝖾(i) query is ${\mathrm{𝖲𝖠}}_{S}\left[i^{\prime }\right]+k$.

The ${\mathrm{𝖨𝖲𝖠}}_{S}\left[i\right]$ access required for extract queries is supported in a similar way. We find the lcs-position S[i+k] for the smallest k≥0, and map it to the corresponding position R[j] by using 𝖠𝗅𝗂𝗀𝗇(R,S). Then we determine ${\mathrm{𝖨𝖲𝖠}}_{R}[j+1]$ by using the reference index, and map it back to ${\mathrm{𝖨𝖲𝖠}}_{S}[i+k+1]$ with $\mathrm{𝖠𝗅𝗂𝗀𝗇}({\mathrm{𝖡𝖶𝖳}}_{R},{\mathrm{𝖡𝖶𝖳}}_{S})$. Finally, we iterate ${\mathrm{𝖡𝖶𝖳}}_{S}$k+1 steps backward with LF queries to find ${\mathrm{𝖨𝖲𝖠}}_{S}\left[i\right]$.

If the target sequence contains long insertions not present in the reference, we may also want to include some SA and ISA samples for querying those regions.

### 3.4. Finding a bwt-invariant subsequence

With the basic relative FMI, we approximate the longest common subsequence of ${\mathrm{𝖡𝖶𝖳}}_{R}$ and ${\mathrm{𝖡𝖶𝖳}}_{S}$ by partitioning the BWTs according to lexicographic contexts, finding the longest common subsequence for each pair of substrings in the partitioning, and concatenating the results. The algorithm is fast, easy to parallelize and quite space-efficient. As such, RFM construction is practical, having been tested with datasets of hundreds of gigabytes in size.

In the following, we describe a more space-efficient variant of the original algorithm [44] for finding a bwt-invariant subsequence. We

- save space by simulating the *mutual SA*${\mathrm{𝖲𝖠}}_{\mathrm{RS}}$ with ${\mathrm{𝖢𝖲𝖠}}_{R}$ and ${\mathrm{𝖢𝖲𝖠}}_{S}$;
- *match* suffixes of R and S only if they are adjacent in ${\mathrm{𝖲𝖠}}_{\mathrm{RS}}$; and
- run-length encode the match arrays to save space.

**Definition 2**

*Let*R*and*S*be two sequences*, *and let*$\mathrm{𝖲𝖠}={\mathrm{𝖲𝖠}}_{\mathrm{RS}}$*and*$\mathrm{𝖨𝖲𝖠}={\mathrm{𝖨𝖲𝖠}}_{\mathrm{RS}}$. *The* left match *of suffix*R[i,∣R∣]*is the suffix*S[𝖲𝖠[𝖨𝖲𝖠[i]−1]−∣R∣,∣S∣], *if*𝖨𝖲𝖠[i]>1*and*𝖲𝖠[𝖨𝖲𝖠[i]−1]*points to a suffix of*S (𝖲𝖠[𝖨𝖲𝖠[i]−1]>∣R∣). *The* right match *of suffix*R[i,∣R∣]*is the suffix*S[𝖲𝖠[𝖨𝖲𝖠[i]+1]−∣R∣,∣S∣], *if*𝖨𝖲𝖠[i]<∣RS∣*and*𝖲𝖠[𝖨𝖲𝖠[i]+1]*points to a suffix of*S.

We simulate the mutual SA ${\mathrm{𝖲𝖠}}_{\mathrm{RS}}$ with ${\mathrm{𝖢𝖲𝖠}}_{R}$, ${\mathrm{𝖢𝖲𝖠}}_{S}$, and the *merging bitvector*$B_{R,S}$ of length ∣RS∣. We set $B_{R,S}\left[i\right]=1$, if ${\mathrm{𝖲𝖠}}_{\mathrm{RS}}\left[i\right]$ points to a suffix of S. The merging bitvector can be built in $𝖮(|S|\cdot t_{\mathrm{𝖫𝖥}})$ time, where $t_{\mathrm{𝖫𝖥}}$ is the time required for an LF query, by extracting S from ${\mathrm{𝖢𝖲𝖠}}_{S}$ and backward searching for it in ${\mathrm{𝖢𝖲𝖠}}_{R}$ [64]. Suffix R[i,∣R∣] has a left (right) match, if $B_{R,S}[{\mathrm{𝗌𝖾𝗅𝖾𝖼𝗍}}_{0}(B_{R,S},{\mathrm{𝖨𝖲𝖠}}_{R}\left[i\right])\;-1]\;=1$ ($B_{R,S}[{\mathrm{𝗌𝖾𝗅𝖾𝖼𝗍}}_{0}(B_{R,S},{\mathrm{𝖨𝖲𝖠}}_{R}\left[i\right])+1]=1)$).

Our next step is building the *match arrays*𝗅𝖾𝖿𝗍 and 𝗋𝗂𝗀𝗁𝗍, which correspond to the arrays A[·][2] and A[·][1] in the original algorithm. This is done by traversing ${\mathrm{𝖢𝖲𝖠}}_{R}$ backwards from ${\mathrm{𝖨𝖲𝖠}}_{R}[|R|]=1$ with LF queries and following the left and the right matches of the current suffix. During the traversal, we maintain the invariant $j={\mathrm{𝖲𝖠}}_{R}\left[i\right]$ with $\left(i,j)\leftarrow ({\mathrm{𝖫𝖥}}_{R}\left(i\right),j-1\right)$. If suffix R[j,∣R∣] has a left (right) match, we use the shorthand $l\left(j\right)={\mathrm{𝗋𝖺𝗇𝗄}}_{1}(B_{R,S},{\mathrm{𝗌𝖾𝗅𝖾𝖼𝗍}}_{0}(B_{R,S},i)-1)$ ($r\left(j\right)={\mathrm{𝗋𝖺𝗇𝗄}}_{1}(B_{R,S},{\mathrm{𝗌𝖾𝗅𝖾𝖼𝗍}}_{0}(B_{R,S},i)+1)$) to refer to its position in ${\mathrm{𝖢𝖲𝖠}}_{S}$.

We say that suffixes R[j,∣R∣] and R[j+1,∣R∣] have the same left match if $l\left(j\right)={\mathrm{𝖫𝖥}}_{S}(l(j+1))$. Let R[j,∣R∣] to R[j+ℓ,∣R∣] be a maximal run of suffixes having the same left match, with suffixes R[j,∣R∣] to R[j+ℓ−1,∣R∣] starting with the same characters as their left matches.^5^ We find the left match of suffix R[j,∣R∣] as $j^{\prime }={\mathrm{𝖲𝖠}}_{S}[l\left(j\right)]$ by using ${\mathrm{𝖢𝖲𝖠}}_{S}$, and set $\mathrm{𝗅𝖾𝖿𝗍}[j,j+\ell -1]=[j^{\prime },j^{\prime }+\ell -1]$. The right match array 𝗋𝗂𝗀𝗁𝗍 is built in a similar way.

The match arrays require 2∣R∣log∣S∣ bits of space. If sequences R and S are similar, the runs in the arrays tend to be long. Hence, we can run-length encode the match arrays to save space. The traversal takes $𝖮(|R|\cdot (t_{\mathrm{𝖫𝖥}}+t_{\mathrm{𝗋𝖺𝗇𝗄}}+t_{\mathrm{𝗌𝖾𝗅𝖾𝖼𝗍}})\;+\mathrm{rd}\cdot t_{\mathrm{𝖫𝖥}})$ time, where $t_{\mathrm{𝗋𝖺𝗇𝗄}}$ and $t_{\mathrm{𝗌𝖾𝗅𝖾𝖼𝗍}}$ denote the time required by rank and select operations, r is the number of runs in the two arrays, and d is the SA sample interval in ${\mathrm{𝖢𝖲𝖠}}_{S}$.^6^

The final step is determining the bwt-invariant subsequence. We find a binary sequence $B_{R}[1,|R|]$, which marks the common subsequence in R, and a strictly increasing integer sequence Y, which contains the positions of the common subsequence in S. This can be done by finding the longest increasing subsequence over R, where we consider both 𝗅𝖾𝖿𝗍[i] and 𝗋𝗂𝗀𝗁𝗍[i] as candidates for the value at position i, and using the found subsequence as Y. If Y[j] comes from 𝗅𝖾𝖿𝗍[i] (𝗋𝗂𝗀𝗁𝗍[i]), we set $B_{R}\left[i\right]=1$, and align suffix R[i,∣R∣] with its left (right) match S[Y[j],∣S∣] in the bwt-invariant subsequence. We can find $B_{R}$ and Y in 𝖮(∣R∣log∣R∣) time with 𝖮(∣R∣log∣R∣) bits of additional working space with a straightforward modification of the dynamic programming algorithm for finding the longest increasing subsequence. The dynamic programming tables can be run-length encoded, but we found that this did not yield good time/space trade-offs.

As sequence Y is strictly increasing, we can convert it into binary sequence $B_{S}[1,|S|]$, marking $B_{S}[Y\left[j\right]]=1$ for all j. Afterwards, we consider the binary sequences $B_{R}$ and $B_{S}$ as the lcs-bitvectors 𝖠𝗅𝗂𝗀𝗇(R,S). Because every suffix of R starts with the same character as its matches stored in the 𝗅𝖾𝖿𝗍 and 𝗋𝗂𝗀𝗁𝗍 arrays, subsequences $R\left[B_{R}\right]$ and $S\left[B_{S}\right]$ are identical.

For any i, let $i_{R}={\mathrm{𝗌𝖾𝗅𝖾𝖼𝗍}}_{1}(B_{R},i)$ and $i_{S}={\mathrm{𝗌𝖾𝗅𝖾𝖼𝗍}}_{1}(B_{S},i)$ be the lcs-positions of rank i. As suffixes $R[i_{R},|R|]$ and $S[i_{S},|S|]$ are aligned in the bwt-invariant subsequence, they are also adjacent in the mutual SA ${\mathrm{𝖲𝖠}}_{\mathrm{RS}}$. Hence,

$${\mathrm{𝖨𝖲𝖠}}_{R}\left[i_{R}\right]<{\mathrm{𝖨𝖲𝖠}}_{R}\left[j_{R}\right]\;⟺\;{\mathrm{𝖨𝖲𝖠}}_{S}\left[i_{S}\right]<{\mathrm{𝖨𝖲𝖠}}_{S}\left[j_{S}\right]$$

for 1≤i,j≤∣Y∣, which is equivalent to the condition in Definition 1. We can convert 𝖠𝗅𝗂𝗀𝗇(R,S) to $\mathrm{𝖠𝗅𝗂𝗀𝗇}({\mathrm{𝖡𝖶𝖳}}_{R},{\mathrm{𝖡𝖶𝖳}}_{S})$ in $𝖮((|R|+|S|)\cdot t_{\mathrm{𝖫𝖥}})$ time by traversing ${\mathrm{𝖢𝖲𝖠}}_{R}$ and ${\mathrm{𝖢𝖲𝖠}}_{S}$ backwards. The resulting subsequence of ${\mathrm{𝖡𝖶𝖳}}_{R}$ and ${\mathrm{𝖡𝖶𝖳}}_{S}$ is bwt-invariant.

Note that the full relative FMI is more limited than the basic index, because it does not handle *substring moves* very well. Let R=xy and S=yx, for two random sequences x and y of length n/2 each. Because ${\mathrm{𝖡𝖶𝖳}}_{R}$ and ${\mathrm{𝖡𝖶𝖳}}_{S}$ are very similar, we can expect to find a common subsequence of length almost n. On the other hand, the length of the longest bwt-invariant subsequence is around n/2, because we can either match the suffixes of x or the suffixes of y in R and S, but not both.

## 4. RELATIVE SUFFIX TREE

The RST is a CST-NPR of the target sequence relative to a CST of the reference sequence. It consists of two major components: the relative FMI with full functionality and the *relative*LCP (RLCP) *array*. The optional relative select structure can be generated or loaded from disk to speed up algorithms based on forward searching. The RLCP array is based on RLZ parsing, while the support for nsv/psv/rmq queries is based on a minima tree over the phrases.

### 4.1. Relative LCP array

Given LCP array 𝖫𝖢𝖯[1,n], we define the *differential*LCP*array*𝖣𝖫𝖢𝖯[1,n] as 𝖣𝖫𝖢𝖯[1]=𝖫𝖢𝖯[1] and 𝖣𝖫𝖢𝖯[i]=𝖫𝖢𝖯[i]−𝖫𝖢𝖯[i−1] for i>1. If $\mathrm{𝖡𝖶𝖳}[i,j]=c^{j+1-i}$ for some c∈Σ, then 𝖫𝖢𝖯[𝖫𝖥(i)+1,𝖫𝖥(j)] is the same as 𝖫𝖢𝖯[i+1,j], with each value incremented by 1 [6]. This means 𝖣𝖫𝖢𝖯[𝖫𝖥(i)+2,𝖫𝖥(j)]=𝖣𝖫𝖢𝖯[i+2,j], making the DLCP array of a repetitive text compressible with grammar-based compression [13].

We make a similar observation in the relative setting. If target sequence S is similar to the reference sequence R, then their LCP arrays should also be similar. If there are long identical ranges ${\mathrm{𝖫𝖢𝖯}}_{R}[i,i+k]={\mathrm{𝖫𝖢𝖯}}_{S}[j,j+k]$, the corresponding DLCP ranges ${\mathrm{𝖣𝖫𝖢𝖯}}_{R}[i+1,i+k]$ and ${\mathrm{𝖣𝖫𝖢𝖯}}_{S}[j+1,j+k]$ are also identical. Hence, we can use RLZ parsing to compress either the original LCP array or the DLCP array.

While the identical ranges are a bit longer in the LCP array, we opt to compress the DLCP array, because it behaves better when there are long repetitions in the sequences. In particular, assembled genomes often have long runs of character N, which correspond to regions of very large LCP values. If the runs are longer in the target sequence than in the reference sequence, the RLZ parsing of the LCP array will have many mismatch characters. The corresponding ranges in the DLCP array typically consist of values {−1,0,1}, making them much easier to compress.

We consider DLCP arrays as strings over an integer alphabet and create an RLZ parsing of ${\mathrm{𝖣𝖫𝖢𝖯}}_{S}$ relative to ${\mathrm{𝖣𝖫𝖢𝖯}}_{R}$. After parsing, we switch to using ${\mathrm{𝖫𝖢𝖯}}_{R}$ as the reference. The reference is stored in a structure we call slarray, which is a variant of LCP-byte. [51]. Small values ${\mathrm{𝖫𝖢𝖯}}_{R}\left[i\right]<255$ are stored in a byte array, while large values ${\mathrm{𝖫𝖢𝖯}}_{R}\left[i\right]\geq 255$ are marked with a 255 in the byte array and stored separately. To quickly find the large values, we also build a ${\mathrm{𝗋𝖺𝗇𝗄}}_{255}$ structure over the byte array. The slarray provides reasonably fast random access and fast sequential access to the underlying array.

The RLZ parsing produces a sequence of phrases $w_{i}\;=(p_{i},\ell_{i},c_{i})$ (see Section 2.3; since we are using Cox et al.’s version, $c_{i}$ is now a string). Because some queries involve decompressing an entire phrase, we limit the maximum phrase length to 1024. We also require that $|c_{i}|>0$ for all i, using the last character of the copied substring as a mismatch if necessary.

Phrase lengths are encoded in the $W_{\ell }$ bitvector in the usual way. We convert the strings of mismatching DLCP values $c_{i}$ into strings of absolute LCP values, append them into the mismatch array $W_{c}$ and store the array as an slarray. The mismatch values are used as *absolute samples* for the differential encoding.

To access ${\mathrm{𝖫𝖢𝖯}}_{S}\left[j\right]$, we determine the phrase $w_{i}$ as usual, and check whether we should return a mismatch character. If so, we compute which one using a prefix sum query on L, and return it. If not, we determine the starting positions $p_{i}$ and $s_{i}$ of the phrase $w_{i}$ in the reference and the target, respectively. We can then compute the solution as

$$\begin{array}{ccc} {\mathrm{𝖫𝖢𝖯}}_{S}\left[j\right] & = & {\mathrm{𝖫𝖢𝖯}}_{S}[s_{i}-1]+\overset{j}{\underset{k=s_{i}}{\sum }}{\mathrm{𝖣𝖫𝖢𝖯}}_{S}\left[k\right]\\ & = & {\mathrm{𝖫𝖢𝖯}}_{S}[s_{i}-1]+\overset{j^{\prime }}{\underset{k=p_{i}}{\sum }}{\mathrm{𝖣𝖫𝖢𝖯}}_{R}\left[k\right]\\ & = & {\mathrm{𝖫𝖢𝖯}}_{S}[s_{i}-1]+{\mathrm{𝖫𝖢𝖯}}_{R}\left[j^{\prime }\right]-{\mathrm{𝖫𝖢𝖯}}_{R}[p_{i}-1], \end{array}$$

where $j^{\prime }=p_{i}+j-s_{i}$. Each RLZ phrase ends with at least one mismatch character, so ${\mathrm{𝖫𝖢𝖯}}_{S}[s_{i}-1]$ is readily available. After finding ${\mathrm{𝖫𝖢𝖯}}_{S}\left[j\right]$, accessing ${\mathrm{𝖫𝖢𝖯}}_{S}[j-1]$ and ${\mathrm{𝖫𝖢𝖯}}_{S}[j+1]$ is fast, as long as we do not cross phrase boundaries.

**Figure 1.**
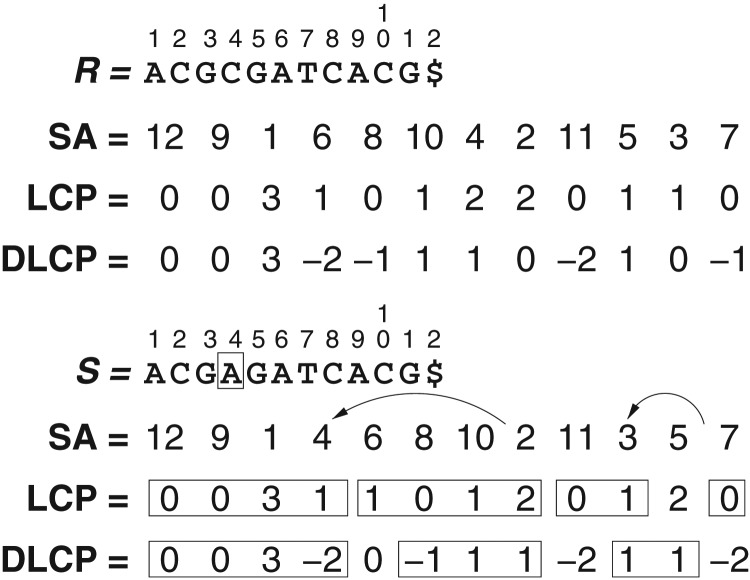
An example of our 𝖱𝖫𝖹 compression of 𝖣𝖫𝖢𝖯.

*Example*. Figure 1 shows an example reference sequence R and target sequence S, with their corresponding arrays 𝖲𝖠, 𝖫𝖢𝖯 and 𝖣𝖫𝖢𝖯. The single edit at S[4] with respect to R[4] may affect the positions of suffixes 4 and previous ones in 𝖲𝖠, although in general only a limited number of preceding suffixes are affected. In our example, suffix 4 moves from position 7 in ${\mathrm{𝖲𝖠}}_{R}$ to position 4 in ${\mathrm{𝖲𝖠}}_{S}$, and suffix 3 moves from position 11 in ${\mathrm{𝖲𝖠}}_{R}$ to position 10 in ${\mathrm{𝖲𝖠}}_{S}$. Each suffix that is moved from ${\mathrm{𝖲𝖠}}_{R}\left[i\right]$ to ${\mathrm{𝖲𝖠}}_{S}\left[j\right]$ may alter the values at positions i or i+1 (depending on whether j>i or j<i), as well as j and j+1, of ${\mathrm{𝖫𝖢𝖯}}_{S}$. We have surrounded in rectangles the conserved regions in ${\mathrm{𝖫𝖢𝖯}}_{S}$ (some are conserved by chance). Even some suffixes that are not moved may change their 𝖫𝖢𝖯 values. In turn, each change in ${\mathrm{𝖫𝖢𝖯}}_{S}\left[k\right]$ may change values ${\mathrm{𝖣𝖫𝖢𝖯}}_{S}\left[k\right]$ and ${\mathrm{𝖣𝖫𝖢𝖯}}_{S}[k+1]$.

After the change, we can parse ${\mathrm{𝖣𝖫𝖢𝖯}}_{S}$ into three phrases (with the copied symbols surrounded by rectangles): (1,4,0), (5,3,−2), (6,2,−2), where the latter is formed by chance. We represent this parsing as $W_{c}=\langle 1,0,0\rangle$ (since we store the absolute ${\mathrm{𝖫𝖢𝖯}}_{S}$ values for the mismatches), $W_{\ell }=100001000100$, and $W_{p}=\langle 1,5,6\rangle$ (or rather $W_{r}=\langle 0,-1,-4\rangle$).

Let us compute ${\mathrm{𝖫𝖢𝖯}}_{S}\left[j\right]$ for j=8. This corresponds to phrase number $i=\mathrm{𝗋𝖺𝗇𝗄}(W_{\ell },j)=2$, which starts at position $s_{i}=\mathrm{𝗌𝖾𝗅𝖾𝖼𝗍}(W_{\ell },i)=6$ in ${\mathrm{𝖫𝖢𝖯}}_{S}$. The corresponding position in ${\mathrm{𝖫𝖢𝖯}}_{R}$ is $p_{i}=W_{p}\left[i\right]=5$ (or rather $p_{i}=s_{i}+W_{r}\left[i\right]=5$), and the mapped position j is $j^{\prime }=p_{i}+j-s_{i}=7$. Finally, ${\mathrm{𝖫𝖢𝖯}}_{S}[s_{i}-1]=W_{c}[i-1]=1$. According to our formula, then, we have ${\mathrm{𝖫𝖢𝖯}}_{S}\left[8\right]={\mathrm{𝖫𝖢𝖯}}_{S}[s_{i}-1]+{\mathrm{𝖫𝖢𝖯}}_{R}\left[j^{\prime }\right]\;-{\mathrm{𝖫𝖢𝖯}}_{R}[p_{i}-1]\;=1+2-1=2$.

### 4.2. Supporting nsv/psv/rmq queries

Suffix tree topology can be inferred from the LCP array with range minimum queries (rmq) and next/previous smaller value (nsv/psv) queries [6]. Some suffix tree operations are more efficient if we also support *next/previous smaller or equal value* (nsev/psev) queries [13]. Query 𝗇𝗌𝖾𝗏(i) (𝗉𝗌𝖾𝗏(i)) finds the next (previous) value smaller than or equal to 𝖫𝖢𝖯[i].

In order to support the queries, we build a 64-ary *minima tree* over the phrases of the RLZ parsing. Each leaf node stores the smallest LCP value in the corresponding phrase, while each internal node stores the smallest value in the subtree. Internal nodes are created and stored in a levelwise fashion, so that each internal node, except perhaps the rightmost one of each level, has 64 children.

We encode the minima tree as two arrays. The smallest LCP values are stored in $M_{\mathrm{𝖫𝖢𝖯}}$, which we encode as an slarray. Plain array $M_{L}$ stores the starting offset of each level in $M_{\mathrm{𝖫𝖢𝖯}}$, with the leaves stored starting from offset $M_{L}\left[1\right]=1$. If i is a minima tree node located at level j, the corresponding minimum value is $M_{\mathrm{𝖫𝖢𝖯}}\left[i\right]$, the parent of the node is $M_{L}[j+1]\;+\lfloor (i-M_{L}\left[j\right])/64\rfloor$, and its first child is $M_{L}[j-1]+64\;\cdot (i-M_{L}\left[j\right])$.

A range minimum query 𝗋𝗆𝗊(sp,ep) starts by finding the minimal range of phrases $w_{l},\ldots ,w_{r}$ covering the query and the maximal range of phrases $w_{l^{\prime }},\ldots ,w_{r^{\prime }}$ contained in the query (note that $l\leq l^{\prime }\leq l+1$ and $r-1\leq r^{\prime }\leq r$). We then use the minima tree to find the leftmost minimum value $j=M_{\mathrm{𝖫𝖢𝖯}}\left[k\right]$ in $M_{\mathrm{𝖫𝖢𝖯}}[l^{\prime },r^{\prime }]$, and find the leftmost occurrence 𝖫𝖢𝖯[i]=j in phrase $w_{k}$. If $l<l^{\prime }$ and $M_{\mathrm{𝖫𝖢𝖯}}\left[l\right]\leq j$, we decompress phrase $w_{l}$ and find the leftmost minimum value $\mathrm{𝖫𝖢𝖯}\left[i^{\prime }\right]=j^{\prime }$ (with $i^{\prime }\geq \mathrm{sp}$) in the phrase. If $j^{\prime }\leq j$, we update $\left(i,j)\leftarrow (i^{\prime },j^{\prime }\right)$. Finally, we check phrase $w_{r}$ in a similar way, if $r>r^{\prime }$ and $M_{\mathrm{𝖫𝖢𝖯}}\left[r\right]<j$. The answer to the range minimum query is 𝖫𝖢𝖯[i]=j, so we return (i,j).^7^ Finally, the particular case where no phrase is contained in [sp,ep] is handled by sequentially scanning one or two phrases in 𝖫𝖢𝖯.

The remaining queries are all similar to each other. In order to answer query 𝗇𝗌𝗏(i), we start by finding the phrase $w_{k}$ containing position i, and then determining 𝖫𝖢𝖯[i]. Next we scan the rest of the phrase to see whether there is a smaller value 𝖫𝖢𝖯[j]<𝖫𝖢𝖯[i] later in the phrase. If so, we return (j,𝖫𝖢𝖯[j]). Otherwise we traverse the minima tree to find the smallest $k^{\prime }>k$ with $M_{\mathrm{𝖫𝖢𝖯}}\left[k^{\prime }\right]<\mathrm{𝖫𝖢𝖯}\left[i\right]$. We decompress phrase $w_{k^{\prime }}$, find the leftmost position j with 𝖫𝖢𝖯[j]<𝖫𝖢𝖯[i], and return (j,𝖫𝖢𝖯[j]).

## 5. EXPERIMENTS

We have implemented the RST in C++, extending the old relative FMI implementation.^8^ The implementation is based on the *Succinct Data Structure Library* (*SDSL*) 2.0 [65]. Some parts of the implementation have been parallelized using *OpenMP* and the *libstdc++ parallel mode*.

As our reference CSA, we used the *succinct SA* (SSA) [58, 66] implemented using SDSL components. Our implementation is very similar to csa_wt in SDSL, but we needed better access to the internals than what the SDSL interface provides. SSA encodes the BWT as a *Huffman-shaped wavelet tree*, combining fast queries with size close to the *order*-0 *empirical entropy*. This makes it the index of choice for DNA sequences [57]. In addition to the plain SSA with uncompressed bitvectors, we also used SSA-RRR with entropy-compressed bitvectors [62] to highlight the the time-space trade-offs achieved with better compression

We sampled SA in suffix order and ISA in text order. In SSA, the sample intervals were 17 for SA and 64 for ISA. In RFM, we used sample interval 257 for SA and 512 for ISA to handle the regions that do not exist in the reference. The sample intervals for suffix order sampling were primes due to the long runs of character N in the assembled genomes. If the number of long runs of character N in the indexed sequence is even, the lexicographic ranks of almost all suffixes in half of the runs are odd, and those runs are almost completely unsampled. This can be avoided by making the sample interval and the number of runs *relatively prime*.

The experiments were done on a system with two 16-core AMD Opteron 6378 processors and 256 GB of memory. The system was running Ubuntu 12.04 with Linux kernel 3.2.0. We compiled all code with g++ version 4.9.2. We allowed index construction to use multiple threads, while confining the query benchmarks to a single thread. As AMD Opteron uses a *non-uniform memory access* architecture, accessing local memory controlled by the same physical CPU is faster than accessing remote memory controlled by another CPU. In order to ensure that all data structures are in local memory, we set the CPU affinity of the query benchmarks with the taskset utility.

As our target sequence, we used the *maternal haplotypes* of the 1000 *Genomes Project individual NA12878* [67]. As the reference sequence, we used the 1000 Genomes Project version of the *GRCh37 assembly* of the *human reference genome*.^9^ Because NA12878 is female, we also created a reference sequence without chromosome Y.

In the following, a basic FMI is an index supporting only find queries, while a full index also supports locate and extract queries.

### 5.1. Indexes and their sizes

Table 2 lists the resource requirements for building the relative indexes, assuming that we have already built the corresponding non-relative structures for the sequences. As a comparison, building an FMI for a human genome typically takes 16–17 min and 25–26 GB of memory. While the construction of the basic RFM index is highly optimized, the other construction algorithms are just the first implementations. Building the optional rselect structures takes 4 min using two threads and around 730 megabytes (∣R∣+∣S∣ bits) of working space in addition to RFM and rselect.

**Table 2.** Sequence lengths and resources used by index construction for NA12878 relative to the human reference genome with and without chromosome Y. Approx and Inv denote the approximate LCS and the bwt-invariant subsequence, respectively. Sequence lengths are in millions of base pairs, while construction resources are in minutes of wall clock time and gigabytes of memory.

	Sequence length				RFM (basic)		RFM (full)		RST	
ChrY	Reference (M)	Target (M)	Approx (M)	Inv (M)	Time (min)	Memory (GB)	Time (min)	Memory (GB)	Time (min)	Memory (GB)
Yes	3096	3036	2992	2980	1.42	4.41	175	84.0	629	141
No	3036	3036	2991	2980	1.33	4.38	173	82.6	593	142

The sizes of the final indexes are listed in Table 3. The full RFM is over twice the size of the basic index, but still 3.3–3.7 times smaller than the full SSA-RRR and 4.6–5.3 times smaller than the full SSA. The RLCP array is 2.7 times larger than the RFM index with the full human reference and 1.5 times larger with the female reference. Hence having a separate female reference is worthwhile, if there are more than a few female genomes among the target sequences. The optional rselect structure is almost as large as the basic RFM index.

**Table 3.** Various indexes for NA12878 relative to the human reference genome with and without chromosome Y. The total for RST includes the full RFM. Index sizes are in megabytes and in bits per character.

	SSA		SSA-RRR		RFM		RST		
ChrY	Basic	Full	Basic	Full	Basic	Full	RLCP	Total	rselect
Yes	1248 MB	2110 MB	636 MB	1498 MB	225 MB	456 MB	1233 MB	1689 MB	190 MB
	3.45 bpc	5.83 bpc	1.76 bpc	4.14 bpc	0.62 bpc	1.26 bpc	3.41 bpc	4.67 bpc	0.52 bpc
No	1248 MB	2110 MB	636 MB	1498 MB	186 MB	400 MB	597 MB	997 MB	163 MB
	3.45 bpc	5.83 bpc	1.76 bpc	4.14 bpc	0.51 bpc	1.11 bpc	1.65 bpc	2.75 bpc	0.45 bpc

Table 4 lists the sizes of the individual components of the relative FMI. Including the chromosome Y in the reference increases the sizes of almost all relative components, with the exception of $\overset{¯}{\mathrm{𝖠𝗅𝗂𝗀𝗇}}\left({\mathrm{𝖡𝖶𝖳}}_{S}\right)$ and 𝖠𝗅𝗂𝗀𝗇(R,S). In the first case, the common subsequence still covers approximately the same positions in ${\mathrm{𝖡𝖶𝖳}}_{S}$ as before. In the second case, chromosome Y appears in bitvector $B_{R}$ as a long run of 0-bits, which compresses well. The components of a full RFM index are larger than the corresponding components of a basic RFM index, because the bwt-invariant subsequence is shorter than the approximate longest common subsequence (see Table 2).

**Table 4.** Breakdown of component sizes in the RFM index for NA12878 relative to the human reference genome with and without chromosome Y in bits per character.

	Basic RFM		Full RFM	
ChrY	Yes (bpc)	No (bpc)	Yes (bpc)	No (bpc)
**RFM**	**0.62**	**0.51**	**1.26**	**1.11**
$\overset{¯}{\mathrm{𝖠𝗅𝗂𝗀𝗇}}\left({\mathrm{𝖡𝖶𝖳}}_{R}\right)$	0.12	0.05	0.14	0.06
$\overset{¯}{\mathrm{𝖠𝗅𝗂𝗀𝗇}}\left({\mathrm{𝖡𝖶𝖳}}_{S}\right)$	0.05	0.05	0.06	0.06
$\mathrm{𝖠𝗅𝗂𝗀𝗇}({\mathrm{𝖡𝖶𝖳}}_{R},{\mathrm{𝖡𝖶𝖳}}_{S})$	0.45	0.42	0.52	0.45
𝖠𝗅𝗂𝗀𝗇(R,S)	–	–	0.35	0.35
SA samples	–	–	0.12	0.12
ISA samples	–	–	0.06	0.06

Bold values aimed to emphasize the base structure (RFM).

The size breakdown of the RLCP array can be seen in Table 5. Phrase pointers and phrase lengths take space proportional to the number of phrases. As there are more mismatches between the copied substrings with the full human reference than with the female reference, the absolute LCP values take a larger proportion of the total space with the full reference. Shorter phrase length increases the likelihood that the minimal LCP value in a phrase is a large value, increasing the size of the minima tree.

**Table 5.** Breakdown of component sizes in the RLCP array for NA12878 relative to the human reference genome with and without chromosome Y. The number of phrases, average phrase length and the component sizes in bits per character. ‘Parse’ contains $W_{r}$ and $W_{\ell }$, ‘Literals’ contains $W_{c}$ and L, and ‘Tree’ contains $M_{\mathrm{𝖫𝖢𝖯}}$ and $M_{L}$.

ChrY	Phrases (million)	Length	Parse (bpc)	Literals (bpc)	Tree (bpc)	Total (bpc)
Yes	128	23.6	1.35	1.54	0.52	3.41
No	94	32.3	0.97	0.41	0.27	1.65

In order to use relative data structures, we also need to have the reference data structures in memory. The basic SSA used by the basic RFM takes 1283 MB with chromosome Y and 1248 MB without, while the full SSA used by the full RFM takes 2162 MB and 2110 MB, respectively. The reference LCP array used by the RLCP array requires 3862 MB and 3690 MB with and without chromosome Y.

### 5.2. Query times

Average query times for the basic operations can be seen in Tables 6 and 7. The results for LF and Ψ queries in the full FMIs are similar to the earlier ones with basic indexes [63]. Random access to the RLCP array is about 30 times slower than to the LCP array, while sequential access is 10 times slower. The nsv, psv and rmq queries are comparable with 1–2 random accesses to the RLCP array.

**Table 6.** Average query times in microseconds for 10 million random queries in the full SSA, the full SSA-RRR and the full RFM for NA12878 relative to the human reference genome with and without chromosome Y.

ChrY	SSA		SSA-RRR		RFM		rselect
	LF (μs)	Ψ (μs)	LF (μs)	Ψ (μs)	LF (μs)	Ψ (μs)	Ψ (μs)
Yes	0.328	1.048	1.989	2.709	3.054	43.095	5.196
No	0.327	1.047	1.988	2.707	2.894	40.478	5.001

**Table 7.** Query times in microseconds in the LCP array (slarray) and the RLCP array for NA12878 relative to the human reference genome with and without chromosome Y. For the random queries, the query times are averages over 100 million queries. The range lengths for the rmq queries were $16^{k}$ (for k≥1) with probability $0.5^{k}$. For sequential access, we list the average time per position for scanning the entire array.

	LCP array		RLCP array				
ChrY	Random (μs)	Sequential (μs)	Random (μs)	Sequential (μs)	nsv (μs)	psv (μs)	rmq (μs)
Yes	0.054	0.002	1.580	0.024	1.909	1.899	2.985
No	0.054	0.002	1.480	0.017	1.834	1.788	3.078

**Figure 2.**
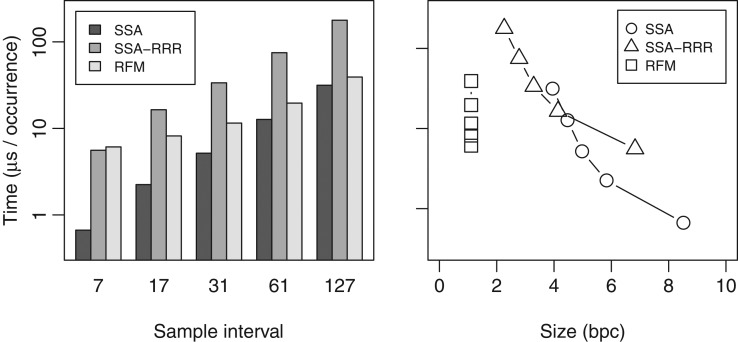
Average find and locate times in microseconds per occurrence for 2 million patterns of length 32 with a total of 255 million occurrences on NA12878 relative to the human reference genome without chromosome Y. Left: query time vs. suffix array sample interval. Right: query time vs. index size in bits per character.

We also tested the locate performance of the full RFM index, and compared it with SSA and SSA-RRR. We built the indexes with SA sample intervals 7, 17, 31, 61 and 127, using the reference without chromosome Y for RFM.^10^ The ISA sample interval was the maximum of 64 and the SA sample interval. We extracted 2 million random patterns of length 32, consisting of characters ACGT, from the target sequence, and measured the total time taken by find and locate queries. The results can be seen in Fig. 2. While SSA and SSA-RRR query times were proportional to the sample interval, RFM used 5.4–7.6 μs per occurrence more than SSA, resulting in slower growth in query times. In particular, RFM with sample interval 31 was faster than SSA with sample interval 61. As the locate performance of the RFM index is based on the sample interval in the reference, it is generally best to use dense sampling (e.g. 7 or 17), unless there are only a few target sequences.

### 5.3. Synthetic collections

In order to determine how the differences between the reference sequence and the target sequence affect the size of relative structures, we built RST for various *synthetic datasets*. We took a 20 MB prefix of the human reference genome as the reference sequence, and generated 25 target sequences with every *mutation rate*p∈{0.0001,0.0003,0.001,0.003,0.01,0.03,0.1}. A total of 90% of the mutations were single-character substitutions, while 5% were insertions and another 5% deletions. The length of an insertion or deletion was k≥1 with probability $0.2\cdot 0.8^{k-1}$.

**Figure 3.**
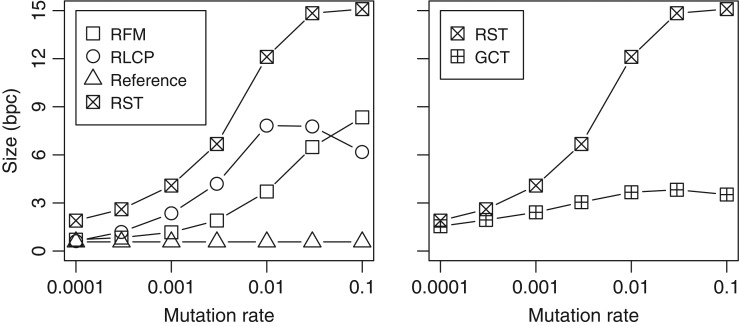
Index size in bits per character vs. mutation rate for 25 synthetic sequences relative to a 20 MB reference.

The results can be seen in Fig. 3 (left). The size of the RLCP array grew quickly with increasing mutation rates, peaking at p=0.01. At that point, the average length of an RLZ phrase was comparable with what could be found in the DLCP arrays of unrelated DNA sequences. With even higher mutation rates, the phrases became slightly longer due to the smaller average LCP values. The RFM index, on the other hand, remained small until p=0.003. Afterwards, the index started growing quickly, eventually overtaking the RLCP array.

We also compared the size of the RST with GCT [15], which is essentially a CST-Sada for repetitive collections. While the structures are intended for different purposes, the comparison shows how much additional space is used for providing access to the suffix trees of individual datasets. We chose to skip the CST-NPR for repetitive collections [13], as its implementation was not stable enough.

Figure 3 (right) shows the sizes of the compressed suffix trees. The numbers for RST include individual indexes for each of the 25 target sequences as well as the reference data, while the numbers for GCT are for a single index containing the 25 sequences. With low mutation rates, RST was not much larger than GCT. The size of RST starts growing quickly at around p=0.001, while the size of GCT stabilizes at 3–4 bpc.

### 5.4. Suffix tree operations

In the final set of experiments, we compared the performance of RST with the SDSL implementations of various CST. We used the maternal haplotypes of NA12878 as the target sequence and the human reference genome without chromosome Y as the reference sequence. We built RST, CST-Sada, CST-NPR and FCST for the target sequence. CST-Sada uses *Sadakane*’*s CSA* (CSA-Sada) [54] as its CSA, while the other SDSL implementations use SSA. We used PLCP as the LCP encoding with both CST-Sada and CST-NPR, and also built CST-NPR with LCP-dac.

We used three algorithms for the performance comparison. The first algorithm is *preorder traversal* of the suffix tree using SDSL iterators (cst_dfs_const_forward_iterator). The iterators use operations 𝖱𝗈𝗈𝗍, 𝖫𝖾𝖺𝖿, 𝖯𝖺𝗋𝖾𝗇𝗍, 𝖥𝖢𝗁𝗂𝗅𝖽 and 𝖭𝖲𝗂𝖻𝗅𝗂𝗇𝗀, though 𝖯𝖺𝗋𝖾𝗇𝗍 queries are rare, as the iterators cache the most recent parent nodes.

The other two algorithms find the *maximal substrings* of the query string occurring in the indexed text, and report the lexicographic range for each such substring. This is a key task in common problems such as computing *matching statistics* [68] or finding *maximal exact matches*. The *forward algorithm* uses 𝖱𝗈𝗈𝗍, 𝖲𝖣𝖾𝗉𝗍𝗁, 𝖲𝖫𝗂𝗇𝗄, 𝖢𝗁𝗂𝗅𝖽 and 𝖫𝖾𝗍𝗍𝖾𝗋, while the *backward algorithm* [69] uses 𝖫𝖥, 𝖯𝖺𝗋𝖾𝗇𝗍 and 𝖲𝖣𝖾𝗉𝗍𝗁.

We used the *paternal haplotypes* of chromosome 1 of NA12878 as the query string in the maximal substrings algorithms. Because some tree operations in the SDSL CST take time proportional to the depth of the current node, we truncated the runs of character N in the query string into a single character. Otherwise searching in the deep subtrees would have made some SDSL suffix trees much slower than RST.

The results can be seen in Table 8. RST was 1.8 times smaller than FCST and several times smaller than the other CST. In depth-first traversal, RST was four times slower than CST-NPR and about 15 times slower than CST-Sada. FCST was orders of magnitude slower, managing to traverse only 5.3% of the tree before the run was terminated after 24 h.

**Table 8.** Compressed suffix trees for the maternal haplotypes of NA12878 relative to the human reference genome without chromosome Y. Component choices; index size in bits per character; average time in microseconds per node for preorder traversal; and average time in microseconds per character for finding maximal substrings shared with the paternal haplotypes of chromosome 1 of NA12878 using forward and backward algorithms. The figures in parentheses are estimates based on the progress made in the first 24 hours.

					Maximal substrings	
CST	CSA	LCP	Size (bpc)	Traversal (μs)	Forward (μs)	Backward (μs)
CST-Sada	CSA-Sada	PLCP	12.33	0.06	79.97	5.14
CST-NPR	SSA	PLCP	10.79	0.23	44.55	0.46
CST-NPR	SSA	LCP-dac	18.08	0.23	29.70	0.40
FCST	SSA	–	4.98	(317.30)	332.80	3.13
RST	RFM	RLCP	2.75	0.90	208.62	3.72
RST + rselect	RFM	RLCP	3.21	0.90	80.20	3.71

It should be noted that the memory access patterns of traversing CST-Sada, CST-NPR and RST are highly local. Traversal times are mostly based on the amount of computation done, while memory latency is less important than in the individual query benchmarks. In RST, the algorithm is essentially the following: (i) compute rmq in the current range; (ii) proceed recursively to the left subinterval and (iii) proceed to the right subinterval. This involves plenty of redundant work, as can be seen by comparing the traversal time (0.90μs per node) to sequential RLCP access (0.017μs per position). A faster algorithm would decompress large parts of the LCP array at once, build the corresponding subtrees in postorder [51], and traverse the resulting trees.

RST with rselect is as fast as CST-Sada in the forward algorithm, 1.8–2.7 times slower than CST-NPR, and 4.1 times faster than FCST. Without the additional structure, RST becomes 2.6 times slower. As expected [69], the backward algorithm is much faster than the forward algorithm. CST-Sada and RST, which combine slow backward searching with a fast tree, have similar performance to FCST, which combines fast searching with a slow tree. CST-NPR is about an order of magnitude faster than the others in the backward algorithm.

## 6. DISCUSSION

We have introduced RST, a new kind of compressed suffix tree for repetitive sequence collections. Our RST compresses the suffix tree of an individual sequence relative to the suffix tree of a reference sequence. It combines an already known relative SA with a novel relative-compressed LCP representation (RLCP). When the sequences are similar enough (e.g. two human genomes), the RST requires about 3 bits per symbol on each target sequence. This is close to the space used by the most space-efficient CST designed to store repetitive collections in a single tree, but the RST provides a different functionality as it indexes each sequence individually. The RST supports query and navigation operations within a few microseconds, which is competitive with the largest and fastest CST.

The size of RST is proportional to the amount of sequence that is present either in the reference or in the target, but not both. This is unusual for relative compression, where any additional material in the reference is generally harmless. Sorting the suffixes in lexicographic tends to distribute the additional suffixes all over the SA, creating many mismatches between the suffix-based structures of the reference and the target. For example, the 60 million suffixes from chromosome Y created 34 million new phrases in the RLZ parse of the DLCP array of a female genome, doubling the size of the RLCP array. Having multiple references (e.g. male and female) can hence be worthwhile when building relative data structures for many target sequences.

While our RST implementation provides competitive time/space trade-offs, there is still much room for improvement. Most importantly, some of the construction algorithms require significant amounts of time and memory. In many places, we have chosen simple and fast implementation options, even though there could be alternatives that require significantly less space without being too much slower.

Our RST is a relative version of the CST-NPR. Another alternative for future work is a relative CST-Sada, using RLZ compressed bitvectors for suffix tree topology and PLCP.

## FUNDING

This work was supported by Basal Funds FB0001, Conicyt, Chile; Fondecyt Grant [1-170048], Chile; Academy of Finland grants [258308] and [250345] (CoECGR); the Jenny and Antti Wihuri Foundation, Finland; and the Wellcome Trust grant [098051].

## Footnotes

1 We refer to ‘long-range’ repetitiveness, where similar texts may be found far away in the text collection.

2 We drop the subscript if the text is evident from the context.

3 For simplicity, we assume that the endmarker is not a part of the bwt-invariant subsequence. Hence 𝖲𝖠[i]>1 for all lcs-positions 𝖡𝖶𝖳[i].

4 If ${\mathrm{𝖡𝖶𝖳}}_{S}\left[i^{\prime }\right]$ and ${\mathrm{𝖡𝖶𝖳}}_{R}\left[j^{\prime }\right]$ are lcs-positions, the corresponding lcs-positions in the original sequences are $S[{\mathrm{𝖲𝖠}}_{S}\left[i^{\prime }\right]-1]$ and $R[{\mathrm{𝖲𝖠}}_{R}\left[j^{\prime }\right]-1]$.

5 The first character of a suffix can be determined by using the 𝖢 array.

6 The time bound assumes text-order sampling.

7 The definition of the query only calls for the leftmost minimum position i. We also return 𝖫𝖢𝖯[i]=j, because suffix tree operations often need it.

8 The current implementation is available at https://github.com/jltsiren/relative-fm.

9
ftp://ftp.1000genomes.ebi.ac.uk/vol1/ftp/technical/reference/

10 With RFM, the sample intervals apply to the reference SSA.
